# Objectively measured physical activity and sedentary time: cross-sectional and prospective associations with adiposity in the Millennium Cohort Study

**DOI:** 10.1136/bmjopen-2015-010366

**Published:** 2016-04-11

**Authors:** Lucy J Griffiths, Francesco Sera, Mario Cortina-Borja, Catherine Law, Andrew Ness, Carol Dezateux

**Affiliations:** 1 Life Course Epidemiology and Biostatistics, UCL Institute of Child Health, London, UK; 2 Clinical Epidemiology, Nutrition and Biostatistics, UCL Institute of Child Health, London, UK; 3 Department of Oral and Dental Science, University of Bristol, Bristol, UK

**Keywords:** adiposity, physical activity, sedentary time, children, cohort studies

## Abstract

**Objective:**

To examine whether physical activity (PA) and sedentary time (ST) in primary school-aged children are associated with adiposity at the start of secondary school, and whether these associations differ by sex or ethnic group.

**Design:**

Nationally representative prospective cohort study.

**Setting:**

Children born across the UK, between 2000 and 2002.

**Participants:**

6497 singleton children.

**Outcome measures:**

Measures of adiposity (body mass index (BMI), fat mass index (FMI) and fat free mass index (FFMI))—obtained at 7 and 11 years.

**Explanatory measures:**

Total daily PA (mean counts per minute (cpm)); minutes of moderate-to-vigorous PA (MVPA); and ST. All assessed at 7 years using accelerometers.

**Results:**

In cross-sectional analyses, total PA was inversely associated with FMI (3.7% (95% CI 2.7% to 4.7%) reduction per 150 cpm increase), as was MVPA (4.2% (CI 3.2% to 5.2%) reduction per 20 min/day increase). Associations were stronger in black and South Asian ethnic groups. Total PA and MVPA were not associated with FFMI. ST was positively associated with FMI (1.3% (CI 0.2% to 2.3%) increase per 50 min/day increase) and inversely associated with FFMI (0.5% (CI 0.2% to 0.7%) reduction per 50 min/day increase). Longitudinally, MVPA at age 7 years remained inversely associated with FMI at age 11 years (1.5% (CI 0.4% to 2.6%) reduction per 20 min/day increase). No association was found between total PA and ST and any of the later adiposity measures.

**Conclusions:**

7-year-old children who are more physically active are less likely to be obese at that age and at age 11 years. These associations were particularly evident in children from black or South Asian ethnicity at age 7 years and in boys at age 11 years. Measurements of fat mass provide valuable insights into ethnic differences in associations between adiposity and activity.

**Strengths and limitations of this study**

- This study used both cross-sectional and prospective study designs to explore concurrent and causal associations between physical activity/sedentary time and adiposity in primary-school aged children in the UK-wide Millennium Cohort.
- Objective measures of physical activity/sedentary time were obtained using accelerometers—however, these may underestimate activities not involving vertical movement of the trunk and aquatic activities; adiposity measures included fat mass and body mass obtained by trained interviewers using standardised protocols.
- Inclusion of children from different ethnic origins enabled associations between activity and adiposity to be explored in these groups.
- Information was available on a range of confounding factors, including puberty, but not other potentially important factors, such as dietary intake.

## Introduction

Recent evidence suggests that the prevalence of childhood obesity is rising around the world, although rates may have stabilised in the past decade in some countries.^1^ ^2^ ^3^ ^4^ Furthermore, the onset of obesity is occurring at ever younger ages.^5^ Mounting evidence points to the ‘potentially devastating’^6^ consequences of this increase; in the short term it has implications for children's health, development and well-being and—in the longer term—for health in young and later adult life. The WHO therefore regards childhood obesity as one of the most serious global public health challenges for the 21st century, reporting that in 2014 there were an estimated 41 million overweight children aged under 5 years alone.^4^

The notion that insufficient physical activity (PA) is a one of the key contributors to obesity is common, and is supported by the logic of the energy-balance equation.^7^ Public health authorities therefore target obesity prevention as a high priority and endeavour to increase time spent being physically active, and decrease time spent being sedentary, across all ages; however, many adults, adolescents and children are reported to be insufficiently active worldwide.^8^ ^9^ ^10^ We have previously reported that only half of 7-year-old children in the UK achieve recommended levels of PA, with significant gender and ethnic variations.^10^ Technological advancements in the assessment of PA and inactivity over the past decade have enabled researchers to estimate levels achieved more accurately, and to investigate associations with a range of outcomes, with overweight and obesity the main focus.

The majority of observational studies that have evaluated cross-sectional associations between adiposity and objectively assessed PA in children suggest that higher activity levels are associated with lower levels of adiposity.^11^ ^12^ ^13^ ^14^ ^15^ However, there is limited evidence on how this varies in children of different ethnic groups.^15^ Cross-sectional associations with sedentary time are less consistent,^12^ ^14^ ^16^ ^17^ although a recent review suggests that sedentary time is usually unrelated to adiposity once activity is taken into account.^18^ Evidence from intervention studies is inconsistent but generally shows no or modest effects of PA programmes on childhood obesity prevention.^19^ ^20^ This may in part reflect the difficulty in changing activity behaviours in childhood.^21^ Few large-scale prospective studies examining both PA and sedentary time have been conducted and, to the best of our knowledge, available evidence for UK children is predominantly from white adolescent populations.^22^ ^23^ ^24^

The main pathway linking PA and sedentary behaviours to overweight and obesity is energy imbalance, resulting from greater energy intake than energy expenditure over time: from a life course perspective one hypothesis relates to a temporal pathway whereby lower energy expenditure related to inactivity leads to greater weight gain. Psychosocial or physical difficulties or socioeconomic disadvantage in childhood may contribute to future risk of increased adiposity via inactivity. Alternatively these factors may confound associations between childhood obesity and PA, by reducing involvement in active pursuits and/or increasing dietary and energy intake.

Our objective was to contribute to this body of research by analysing data from a large UK-wide cohort of primary school-aged children in whom objective assessments of activity (total amount of PA and moderate-to-vigorous physical activity), sedentary time and adiposity (body mass index (BMI), fat mass index (FMI) and fat free mass index (FFMI)) were obtained at 7 years. We analysed cross-sectional (age 7 years) and prospective (activity and sedentary time at age 7 years and adiposity at age 11 years) associations, thereby addressing the need for more evidence on longitudinal associations,^25^ ^26^ while also exploring if these associations differed by sex or ethnic group.

## Methods

### Study participants

We analysed data from the Millennium Cohort Study (MCS), a longitudinal study of children born in the UK between September 2000 and January 2002.^27^ Children living in disadvantaged areas, from ethnic minority groups and from Wales, Scotland and Northern Ireland were over-represented by using a stratified clustered sampling design to ensure the cohort was nationally representative. The first study contact (MCS1) with the cohort child was around age 9 months, when information was collected on 72% of those approached, providing information on 18 818 singleton infants. Five further surveys were carried out when children were aged 3 (MCS2), 5 (MCS3), 7 (MCS4) and 11 (MCS5) years.

Detailed information regarding demographic, social and health factors relating to the children and their families was obtained through home-based interviews. The MCS received ethical approval from the South West Multi-Centre Research Ethics Committee (MCS1), the London Multi-Centre Research Ethics Committee (MCS2 and MCS3), Northern and the Yorkshire Research Ethics Committee (MCS4) and the Yorkshire and Humber Research Ethics Committee (MCS5). The study reported here did not require additional ethics approval and used data from MCS1, MCS4 and MCS5 (as detailed below).

### Exposure measures: PA and sedentary time

PA and sedentary time were assessed objectively using the Actigraph GT1M accelerometer (Actigraph, Pensacola, Florida, USA), a small and lightweight, non-waterproof device, which, in this study, was worn on an elastic belt round the child's waist. The Actigraph GT1M has been extensively validated in children and compares favourably against observational techniques,^28^ heart rate monitoring,^29^ indirect and room calorimetry^30^ ^31^ and doubly labelled water techniques.^32^ It has been shown to be robust when used in other large-scale PA studies in children.^16^ ^33^

A total of 13 681 singleton children participated in MCS4 interviews when they were invited to participate in an accelerometry study, which took place over a 15-month period between May 2008 and August 2009. Those who consented (12 872; 94.5%) were posted an accelerometer, programmed to collect and aggregate data over 15 s epochs. Participants were instructed to start wearing their accelerometer the morning after they received it and to continue doing so for 7 days during waking hours. They were asked to remove the monitor when bathing or swimming. The accelerometer assessments started after the MCS4 interviews had been completed, resulting in a median interval of 36 weeks (IQR 29–45) between the interview and the date accelerometers were worn.

Accelerometers were returned by 9772 singleton children. Data were downloaded using Actigraph software V.3.8.3 (Actigraph, Pensacola, Florida, USA) and processed using the package pawacc^34^ ^35^ for the R statistical computing environment (R Development Core Team. R: A language and environment for statistical computing. R Foundation for Statistical Computing, Vienna, Austria. 2014. http://www.R-project.org/). Non-wear time was defined as any time period of consecutive zero-counts lasting 20 min or more:^15^ these periods were removed from the summation of activity. Data were summarised as counts per minute (cpm) and values for moderate-to-vigorous physical activity ≥11 715 removed from the dataset, based on a reliability study that indicated that count values above this threshold were extreme and likely to be spurious.^36^ Only days with 10 h or more of recorded time were considered as valid and retained in the data set, and only participants with at least two such days were included in the analyses.^37^ We have previously shown high reliability (r = 0.86) of this criteria in the MCS cohort.^37^ The application of these criteria resulted in a sample size of 6497 singleton children. Reliable accelerometer data were less likely to be acquired from children who were: male; overweight/obese; of white, mixed or ‘other’ ethnicity; living in disadvantaged areas; had less educated mothers and/or lone mothers.^38^ However, overall, small differences were found in the demographic characteristics of the children included in our analyses relative to the whole cohort sample interviewed at age 7 years, as detailed previously.^10^

For each child, we derived three exposure variables: average daily minutes of moderate-to-vigorous physical activity (using an accelerometer-defined boundary of ≥2240 counts^39^), average daily minutes spent in sedentary time (using the accelerometer-defined boundary of <100 counts^39^) and total PA, calculated as the average cpm over the full period of valid recording. As average daily minutes spent in PA and sedentary time are related to observed or wear time, these variables were adjusted for standardised wear time across the subjects.^40^

### Outcome measures: adiposity

At the fourth and fifth surveys, measures of body composition were taken by trained interviewers. Body fat measurements were obtained using the Tanita BF-522W scales (Tanita UK Ltd, Middlesex, UK); children were asked to stand bare-footed and without outdoor clothing on the metal sole-plates of the scales, which incorporate electrodes and measure foot-to-foot bioelectrical impedance. Bioelectrical impedance analysis assesses body fat by passing a very small current through the body and assessing differences in impedance caused by the fact that fat and lean tissues have different electrical properties. The interviewers entered information on each child's age, sex and height into the scales, and recorded the resultant calculated estimate of body fat percentage for that child. Height was measured using Leicester Height Measure Stadiometers (Seca Ltd, Birmingham, UK) and recorded to the nearest millimetre with the head in the Frankfort plane. Weight was measured in kilograms to one decimal place using the Tanita BF-522W scales. The height and weight measures were used to derive BMI. FMI and FFMI were calculated using fat mass and fat free mass measures, respectively (box 1).

Box 1 **Adiposity formulas**

Body Mass Index (BMI): weight (kilograms)/height (metres) squared.

Fat mass (kilograms): percent body fat×weight.

Fat free mass (kilograms): weight—fat mass.

Fat Mass Index (FMI): fat mass/height squared.

Fat Free Mass Index (FFMI): fat free mass/height squared.

### Potential confounding variables

Potential confounding factors were identified a priori from existing literature as factors associated with PA and obesity. In order to identify a minimal set of confounders we constructed a direct acyclic graph (DAG)^41^ for the cross-sectional analyses and checked that no over-adjustment was present. This set included the child's gender and ethnicity (the latter categorised according to guidelines from the Office for National Statistics^42^) collected at MCS1; maternal weight status (defined using BMI cut-offs), age at birth of the cohort member, social class (National Statistics Socioeconomic Classification (NS-SEC)^43^) and highest educational attainment; annual household income (quintiles); number of cars or vans in the household that are used regularly; lone parenthood status; number of children in the household; country of residence; index of multiple deprivation; and rural/urban area classification of residence. Unless otherwise stated, information on all of these factors was collected at MCS4. These confounders were also used in prospective analyses.

Information on pubertal status was collected at MCS5, using five parentally reported puberty indicators; three were common between boys and girls—growth spurt, body hair, skin changes—while voice change and facial hair were specific for boys and breast growth and age at menarche specific for girls. To reduce the dimensionality of these covariates we built a categorical puberty indicator. Multiple correspondence analyses^44^ were performed separately for boys and girls. Using the optimal category scores provided by this, we calculated two quantitative scores; a cluster analysis (complete linkage) was then performed using these two scores and three groups of children were identified after a visual analysis of the dendrogram: those in whom puberty had not started, had barely started or was likely to have started.

Other potential confounding factors explored but, based on the DAG criteria, not included were: presence of a long-standing illness or other illness limiting their everyday activity; diagnosis of asthma; psychological well-being; peer relationship problems; paternal weight status; maternal employment status; housing tenure; and type of residence.

### Statistical analysis

Analyses were performed using Stata/SE 13 (Stata Corporation, Texas, USA). Considering the stratified cluster sampling design of MCS study, weights to adjust for attrition between contacts at successive MCS sweeps and for missing accelerometer data were taken into account during the estimation using the Stata command svyset. Gender and ethnic differences between total PA, sedentary time and moderate-to-vigorous physical activity were assessed adjusting by season of measurement and a weekend/weekday contrast.

Multiple linear regression models were fitted to examine cross-sectional associations between total PA levels, moderate-to-vigorous physical activity and sedentary time and each of the adiposity measures (BMI, FMI and FFMI); the latter variables were log-transformed due to their skewed distributions. For each regression coefficient b, we calculated the quantity 100×(e^b^−1); similarly, the lower and upper bounds of b's 95% CI were subject to the same back-transformation. These values can be interpreted as the percentage change between geometric means of the adiposity measure associated with varying levels of the covariates of interest. The p values were calculated using the command nlcom in Stata, which is based on the δ method^45^ to approximate the distribution of non-linear combinations of parameter estimates. The models were adjusted for child's sex, ethnicity, country of residence and age at measurement; maternal BMI, age, social class and highest academic qualification; and household annual income, number of cars, lone parenthood status, number of children in the household, urban/rural ward of residence.

Linear regression models were also fitted to evaluate prospective associations between total PA levels, moderate-to-vigorous physical activity and sedentary time at age 7 (MCS4) and the three adiposity measures at age 11 (MCS5). Analysis of covariance was used; the baseline value of the adiposity measure was considered as a covariate, alongside the particular exposure variable and confounding variables. Again, adiposity measures were log transformed, and the regression coefficient transformed as described before. A similar strategy was also used to fit the model; adjustment was made for the same variables as in the cross-sectional analyses, in addition to the baseline adiposity measurement and puberty indicator.

In the cross-sectional and prospective analyses, the effects of joint variation between exposures and the child's sex, and ethnicity, were explored by considering an interaction term in the regression models. All regression models were initially fitted in the complete case sample. For the prospective analyses, around 500 (8.2%) children had missing data for outcome variables (adiposity measurements) or covariates (particularly for pubertal status). Multiple imputation analysis was performed to mitigate possible bias due to item non-response. For the cross-sectional analyses five imputed data sets were built using the (weighted) iterative chain algorithm, and 10 for the prospective analyses; estimates were combined using Rubin's rule.^46^

## Results

The majority of children (85%) were white, 51% were boys and 20% overweight or obese at age 7 years. Almost half of mothers were overweight or obese. Sociodemographic measures at ages 7 and 11 years are summarised in table 1 and showed little change over this interval, with the exception of an increase in the prevalence of overweight at age 11 years.

**Table 1:** Sample characteristics

	MCS4 (n=6497)		MCS5 (n=6073)	
Variable	n	Per cent*	n	Per cent*
Child's gender				
Male	3176	50.9	2950	51.1
Female	3321	49.1	3123	48.9
Child's ethnicity†				
White	5685	85.1	5327	85.0
Mixed	167	3.3	153	3.1
South Asian	382	7.1	359	7.3
Black	141	2.9	119	2.7
Other	90	1.6	87	1.9
Missing	32		28	
Obesity (IOTF cut-offs)				
Normal	5345	79.8	4522	73.0
Overweight (not including obese)	836	14.2	1142	21.0
Obese	289	6.0	290	6.0
Missing	27		119	
Puberty indicator				
Puberty had not started			3498	58.8
Puberty had barely started			1772	29.3
Puberty was likely to have started			651	11.9
Missing			152	
Maternal weight status (BMI-defined groups)				
Normal weight	3109	56.1	2929	56.3
Overweight (not including obese)	1547	28.1	1442	27.9
Obese	830	15.8	782	15.8
Missing	1011		920	
Main respondent's age at the birth of the cohort member				
14–19	283	8.8	246	8.9
20–29	2660	45.6	2472	46.4
30+	3554	45.6	3355	44.7
Maternal socioeconomic circumstances				
Managerial and professional occupations	1806	26.1	2082	27.5
Intermediate occupations	922	18.3	1129	18.2
Small employers and own account workers	387	6.9	420	6.8
Lower supervisory and technical occupations	177	5.3	260	5.1
Semiroutine and routine occupations	1104	34.6	1710	35.5
Never worked and long-term unemployed	2101	6.8	241	6.9
Missing	256		231	
Main respondent's highest academic qualification				
Degree	1644	18.0	1580	17.4
Diploma	803	10.0	756	9.7
A/AS/S levels	677	8.8	645	8.8
GCSE grades A-G	2536	44.3	2348	45.1
Other	143	2.7	132	2.9
None of the above	686	16.2	605	16.1
Missing	8		7	
Lone parenthood status				
Non-lone parent	5485	77.3	5169	77.3
Lone parent	989	22.7	884	22.7
Missing	23		20	
Household annual income (quintiles)				
Bottom	960	20.6	859	20.7
Second	1164	20.2	1071	20.7
Third	1382	19.8	1293	20.1
Fourth	1513	20.1	1438	19.9
Top	1477	19.3	1411	18.6
Missing	1		1	
Number of cars or vans in the household (regular use)				
0	579	14.3	513	14.4
1	2304	37.1	2133	37.6
2	3230	42.9	3068	42.3
3	374	5.7	353	5.7
Missing	10		6	
Number of children in the household				
1	719	12.2	670	12.2
2	3131	46.6	2942	46.0
3	1820	27.8	1704	28.2
4+	812	13.4	744	13.6
Missing	15		13	
Country of residence				
England	4201	81.6	3933	81.5
Wales	899	5.1	833	5.1
Scotland	761	9.2	710	9.2
Northern Ireland	636	4.1	597	4.2
IMD				
Least deprived	1499	20.4	1435	20.4
Second	1255	19.2	1175	18.6
Third	1255	20.2	1181	20.3
Fourth	1244	18.7	1147	18.9
Most deprived	1243	21.5	1134	21.8
Missing	1		1	
Urban/rural area of residence				
Urban	5480	86.8	5110	86.6
Rural	1016	13.2	962	13.4
Missing	1		1	

*Weighted estimate.

†Collected at the first MCS survey; all other variables were collected in the fourth (age 7 years) survey.

BMI, body mass index; GCSE, General Certificate of Secondary Education; IMD, index of multiple deprivation; IOTF, International Obesity Task Force; MCS, Millennium Cohort Study.

As reported previously,^10^ boys were more active and less sedentary than girls at age 7 years. They were also taller and had lower adiposity as assessed by lower mean fat mass and higher fat free mass (table 2). By age 11 years, the boys were lighter, shorter and still had lower adiposity (lower body mass and fat mass and higher fat free mass) than girls. As expected, marked sex differences in onset of puberty were seen at age 11 years, with 1 in 5 girls, but less than 1 in 20 boys, having started puberty by this age.

**Table 2:** Mean and SD of the anthropometric and physical activity variables by sex and MCS survey

	Girls (n=3321)			Boys (n=3176)			
	n	Mean	SD	n	Mean	SD	p Value*
MCS4							
Weight (kg)	3315	25.4	5.0	3161	25.7	5.0	0.2
Height (cm)	3319	123.3	5.7	3172	124.1	5.4	<0.001
BMI (kg/m^2^)	3312	16.6	2.4	3158	16.6	2.3	0.6
FMI (kg/m^2^)	3281	3.8	1.6	3119	3.4	1.6	<0.001
FFMI (kg/m^2^)	3281	12.8	1.0	3118	13.1	1.0	<0.001
Total PA (daily cpm)†	3321	573.9	146.4	3176	644.8	155.4	<0.001
Daily sedentary time (min)†	3321	398.7	50.5	3176	382.2	49.4	<0.001
Daily MVPA (min)†	3321	56.4	19.9	3176	69.9	23.0	<0.001
	Girls (n=3123)			Boys (n=2950)			
MCS5							
Weight (kg)	3043	42.1	10.4	2880	40.6	9.8	<0.001
Height (cm)	3080	146.8	7.8	2910	145.8	6.7	<0.001
BMI (kg/m^2^)	3056	19.4	3.7	2898	19.0	3.7	0.003
FMI (kg/m^2^)	3002	5.0	2.5	2834	4.0	2.5	<0.001
FFMI (kg/m^2^)	3002	14.4	1.4	2834	14.9	1.5	<0.001
**Puberty indicator**	n	Per cent		n	Per cent		
Puberty had not started	1054	32.9		2444	83.7		
Puberty had barely started	1433	46.4		339	12.8		
Puberty was likely to have started	553	20.7		98	3.5		<0.001

*The p values indicate differences between genders; obtained from an adjusted Wald test with girls as the referent.

†Adjusted by season of measurement and weekend/weekday.

BMI, body mass index; cpm, counts per minute; FFMI, fat free mass index; FMI, fat mass index; MCS, Millennium Cohort Study; Min, minutes; MVPA, moderate-to-vigorous physical activity; PA, physical activity.

Children of South Asian ethnic origin were less active and had higher fat mass and lower fat free mass than white children (table 3). In contrast, black children were more active but also had higher body and fat mass. By age 11 years, these ethnic differences in adiposity persisted. Black girls were twice as likely to have started puberty than white or South Asian girls.

**Table 3:** Description of the anthropometric and physical activity variables at age 7 years by ethnicity

	White (n=5685)		Mixed (n=167)			South Asian (n=382)			Black/black British (n=141)			Other (n=90)		
	Mean	SD	Mean	SD	p Value*	Mean	SD	p Value	Mean	SD	p Value	Mean	SD	p Value
MCS4														
Weight (kg)	25.4	4.7	26.0	5.4	0.3	25.4	5.6	0.95	30.8	6.3	<0.001	24.0	3.6	0.003
Height (cm)	123.5	5.6	124.6	4.9	0.031	124.1	5.1	0.07	127.9	5.0	<0.001	123.3	3.9	0.8
BMI (kg/m^2^)	16.6	2.2	16.6	2.6	0.8	16.3	2.7	0.2	18.7	3.3	<0.001	15.8	1.9	0.001
FMI (kg/m^2^)	3.5	1.4	3.7	1.8	0.3	3.9	2.0	0.023	5.0	2.3	<0.001	3.4	1.3	0.2
FFMI (kg/m^2^)	13.0	1.0	13.0	1.0	0.6	12.4	0.9	<0.001	13.7	1.1	<0.001	12.5	1.0	<0.001
Total PA (daily cpm)†	613.9	157.9	584.4	122.6	0.003	570.7	134.3	<0.001	627.9	134.7	0.3	590.7	174.9	0.3
Daily sedentary time (min)†	389.6	50.7	406.1	47.0	<0.001	391.8	51.6	0.3	388.0	39.8	0.6	393.2	55.2	0.6
Daily MVPA (min)†	63.3	23.0	62.2	17.2	0.3	59.9	19.7	0.009	70.3	22.0	0.012	62.4	23.8	0.7
	White (n=5327)		Mixed (n=153)			South Asian (n=359)			Black/black British (n=119)			Other (n=87)		
MCS5														
Weight (kg)	41.1	10.0	42.1	11.0	0.4	41.6	9.5	0.5	50.8	10.5	<0.001	38.8	6.6	0.046
Height (cm)	146.2	7.4	147.2	6.2	0.098	146.6	6.3	0.4	151.0	6.1	<0.001	145.5	5.0	0.5
BMI (kg/m^2^)	19.1	3.6	19.2	4.0	0.8	19.2	3.6	0.5	22.3	4.3	<0.001	18.3	2.9	0.053
FMI (kg/m^2^)	4.4	2.5	4.6	3.0	0.5	4.9	2.3	0.001	7.0	4.0	0.001	4.5	2.2	0.99
FFMI (kg/m^2^)	14.7	1.4	14.6	1.4	0.6	14.2	1.4	<0.001	15.3	1.9	0.004	14.0	1.1	<0.001
**Puberty indicator**	**n**	**Per cent**	** n**	**Per cent**		**n**	**Per cent**		** n**	**Per cent**		** n**	**Per cent**	
Puberty had not started	3127	59.4	76	45.3		182	60.9		46	44.3		51	72.5	
Puberty had barely started	1566	29.2	49	35.0		87	26.4		42	31.3		20	25.4	
Puberty was likely to have started	548	11.4	25	19.7		44	12.7		27	24.4		5	2.1	<0.001

*The p values indicate differences between ethnic groups; obtained from an adjusted Wald test with white children as the referent.

†Adjusted by season of measurement and weekend/weekday.

BMI, body mass index; cpm, counts per minute; FFMI, fat free mass index; FMI, fat mass index; MCS, Millennium Cohort Study; Min, minutes; MVPA, moderate-to-vigorous physical activity; PA, physical activity.

In adjusted cross-sectional analyses, total PA and moderate-to-vigorous physical activity were inversely associated with indices of body mass and fat mass at age 7 years (table 4). Body mass was on average 0.84% (95% CI 0.45% to 1.22%) and fat mass 3.68% (95% CI 2.68% to 4.68%) lower for each 150 cpm increase in total PA, while body mass was 1.05% (95% CI 0.66% to 1.43%), and fat mass 4.18% (95% CI 3.15% to 5.21%), lower for each additional 20 min period spent in moderate-to-vigorous physical activity. Fat mass was on average 1.27% (0.21% to 2.33%) higher for each additional 50 min period of daily sedentary time.

**Table 4:** Cross-sectional analysis: adjusted percent change in anthropometric indices measured at 7 years for given changes in the summary physical activity or sedentary variables assessed at 7 years

	BMI			FMI			FFMI		
Exposures	Per cent change	95% CI	p Value	Per cent change	95% CI	p Value	Per cent change	95% CI	p Value
Total PA (150 counts increase*)	−0.84	(−1.22 to −0.45)	<0.0001	−3.68	(−4.68 to −2.68)	<0.0001	0.06	(−0.17 to 0.29)	0.62
Sedentary time (50 min increase*)	−0.01	(−0.41 to 0.40)	0.97	1.27	(0.21 to 2.33)	0.019	−0.46	(−0.71 to −0.21)	<0.0001
MVPA (20 min increase*)	−1.05	(−1.43 to −0.66)	<0.0001	−4.18	(−5.21 to −3.15)	<0.0001	−0.04	(−0.27 to 0.18)	0.69

Regression models were adjusted for weekend, season, child's sex, age at measurement, child's ethnicity, maternal BMI, main respondent's age at the birth of the cohort member, maternal socioeconomic circumstances, main respondent's education, household annual income, cars or vans (regular use), lone parenthood status, number of children in the household, Country by (index of multiple deprivation) interaction and urban/rural indicators.

*These values/increments approximate to one SD of these measures.

BMI, body mass index; FFMI, fat free mass index; FMI, fat mass index; Min, minutes; MVPA, moderate-to-vigorous physical activity; PA, physical activity.

**Figure 1:**
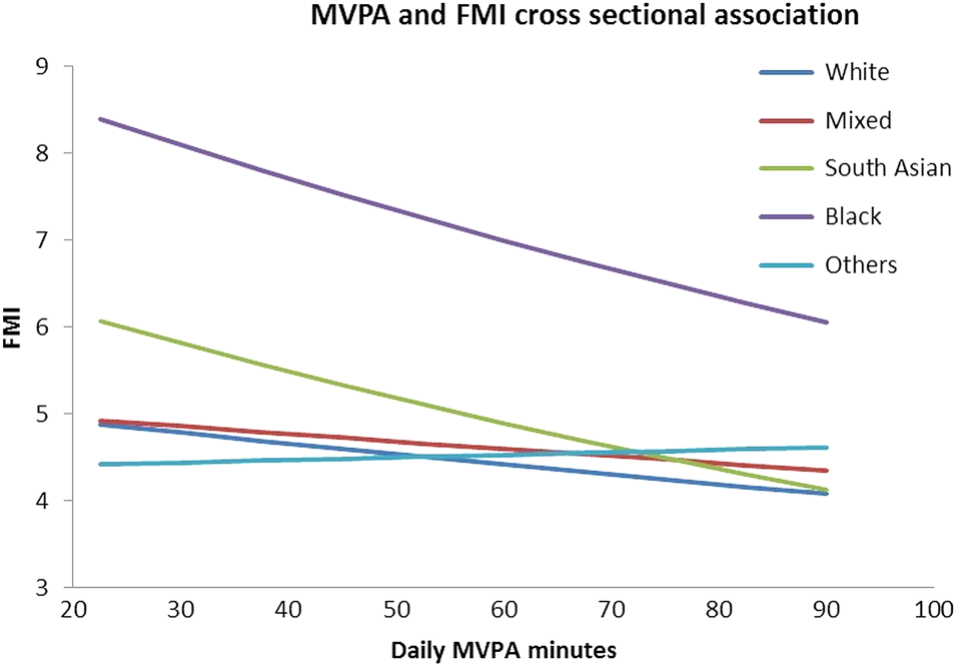
Ethnic differences in strength of cross-sectional associations between MVPA and FMI at age 7 years. FMI, fat mass index; MVPA, moderate-to-vigorous physical activity.

Black children and those of South Asian ethnic origin showed the greatest decrease in fat mass index for each increment in time spent in moderate-to-vigorous physical activity across the range of 20–90 min. Significant interactions with ethnicity were found in cross-sectional analyses between total PA and fat mass (p=0.003) and moderate-to-vigorous physical activity and fat mass (p=0.020); the latter is shown in figure 1. Fat mass was, on average, 12.74% (95% CI 3.97 to 21.51) and 7.44% (95% CI 3.74% to 11.15%) lower per 150 cpm increase in total PA level in black children and those of South Asian ethnic origin respectively and 8.22% (95% CI 5.05% to 11.39%) lower for each additional 20 min of moderate-to-vigorous physical activity in children of South Asian ethnic origin.

Interactions with gender were not significant.

By 11 years, moderate-to-vigorous physical activity but not total PA levels at age 7 years remained inversely associated with body mass and fat mass (table 5). The effect sizes were also smaller than those obtained in the cross-sectional analyses; body mass and fat mass at age 11 years were on average 0.39% (0.04% to 0.74%) and 1.5% (0.44% to 2.58%) lower for each 20 min increase in daily moderate-to-vigorous physical activity at age 7 years.

**Table 5:** Longitudinal analysis: adjusted percent change in anthropometric indices measured at 11 years for given changes in the summary physical activity or sedentary variables assessed at 7 years

	BMI			FMI			FFMI		
Exposures	Per cent change	95% CI	p Value	Per cent change	95% CI	p Value	Per cent change	95% CI	p Value
Total PA (150 counts increase*)	−0.15	(−0.50 to 0.20)	0.40	−0.94	(−2.10 to 0.28)	0.11	0.05	(−0.17 to 0.27)	0.64
Sedentary time (50 min increase*)	−0.25	(−0.59 to 0.09)	0.16	−0.25	(−1.36 to 0.85)	0.65	−0.16	(−0.38 to 0.05)	0.13
MVPA (20 min increase*)	−0.39	(−0.74 to −0.04)	0.029	−1.51	(−2.58 to −0.44)	0.006	−0.06	(−0.27 to 0.14)	0.53

Regression models were adjusted for weekend, season, interaction between child's sex and puberty indicators, baseline anthropometric index, age at measurement, child's ethnicity, maternal BMI, main respondent's age at the birth of the cohort member, maternal socioeconomic circumstances, main respondent's education, household annual income, cars or vans (regular use), lone parenthood status, number of children in the household, Country by (index of multiple deprivation) interaction and urban/rural indicators.

*These values/increments approximate one SD of these measures.

BMI, body mass index; FFMI, fat free mass index; FMI, fat mass index; Min, minutes; MVPA, moderate-to-vigorous physical activity; PA, physical activity.

In this longitudinal analysis, we also found a significant interaction for sex (p<0.001), where the association between moderate-to-vigorous physical activity and body mass or fat mass was present only in boys: body mass and fat mass at age 11 were on average 2.53% (0.88% to 4.17%) and 2.89% (1.34% to 4.40%) lower for each 20 min increase in daily moderate-to-vigorous physical activity at age 7 years. There were no significant interactions with ethnicity.

## Discussion

### Statement of the principal findings

Using data from a large nationally representative sample of primary school-aged children, we found that children who were less active at age 7 years have higher levels of adiposity, as assessed using indices of body mass and fat mass. These associations were particularly marked with the measure of fat mass but not body mass for children of black and South Asian ethnic origin. Children who were more sedentary were also likely to have greater fat mass at 7 years. In longitudinal analyses, boys (but not girls) who spent more time in moderate-to-vigorous physical activity at age 7 years had less body or fat mass—at age 11 years, but there was no association with total activity levels or being sedentary at 7 years and adiposity at 11 years.

### Comparison with the literature

Our findings from cross-sectional analyses are consistent with other large population-based studies.^12^ ^13^ ^14^ ^15^ Ekelund *et al*^12^ reported an inverse association between total PA and fat mass, assessed using the sum of five skinfold measures, in 9–10 year old children across four European centres. Evidence from the Avon Longitudinal Study of Parents and Children (ALSPAC) also suggests an inverse association between total and moderate-to-vigorous physical activity, and fat mass assessed through dual-energy X-ray absorptiometry measurements in 12-year-olds.^13^ Other studies also report similar inverse associations in 9–10 year olds.^14^ ^15^ These studies acknowledge the limitations of cross sectional analyses, potential reverse causality and the importance of and need for prospective that is, longitudinal studies.

Within our prospective longitudinal study, in boys moderate or vigorous activity at age 7 years remained inversely associated with both body mass and fat mass measured 4 years later. This is to the best of our knowledge the first time this has been reported in a large scale population-based study of primary school-aged children.

In our study, associations between total PA levels and sedentary time with subsequent adiposity were not significant in the prospective analyses but a longitudinal effect was still evident for moderate-to-vigorous physical activity. Findings from other studies also suggest that activity of vigorous intensity may be more strongly associated with adiposity outcomes than activity or lower intensity or total PA,^14^ ^47^ although the mechanisms underlying more beneficial effects of more vigorous activity remain unknown. for example, Fisher *et al*^47^ reported that activity of moderate-to-vigorous intensity was significantly associated with follow-up FMI independent of total PA or sedentary time in a smaller study of 280 9–10-year-old children. By contrast, other published studies in this age group have reported no prospective associations between activity and adiposity.^24^ ^48^

Our findings are however also consistent with those of prospective studies conducted in adolescent populations. Pate *et al*^49^ identified five studies reporting inverse associations between total activity levels or moderate-to-vigorous physical activity measured at baseline and fat mass at intervals of 1–7 years of follow-up. Within ALSPAC, higher levels of PA at age 12 years were associated with lower levels of fat mass at age 14 years.^22^ Basterfield *et al*^50^ reported that changes in moderate-to-vigorous physical activity were associated with changes in fat mass over a 2-year period: interestingly they noted an interaction and that this finding was only present in boys—consistent with our finding. Although our observed effect sizes were small, increasing activity levels in boys may have important implications at the population level in the prevention of excess adiposity. We propose that our finding of a significant association in boys but not girls may reflect differences in tracking of PA levels^51^ and/or in dietary behaviours by sex which, in turn, could also be affected by differences in age of onset of puberty in boys and girls. However, the data available precluded exploring these hypotheses.

Evidence on associations between PA and adiposity by ethnic origin is limited and inconsistent: studies from the USA report differences between black and white adolescent girls,^52^ while a cross-sectional study from the UK reports that they are broadly similar across those of South Asian, black African-Caribbean and white European origin.^15^ In contrast, we observed stronger associations between activity and adiposity in children of South Asian and black origin; however, interactions between ethnicity and PA were only observed within the cross-sectional analyses. Associations with ethnicity, and possible underlying mechanisms, warrant further investigation given the established differences in activity levels,^10^ ^53^ risk of adiposity and markers of cardiovascular risk^54^ in children from different ethnic groups.

A growing body of research has explored associations between sedentary time and adiposity markers, including fat and body mass. We found that sedentary time was positively associated with FMI, but only within the cross-sectional analysis. A positive association between sedentary time and markers of adiposity is supported by some^55^ ^56^ but not by other studies;^12^ ^14^ ^16^ ^23^ ^24^ ^57^ ^58^ significant associations are frequently reduced or removed completely following adjustment for levels of PA.^18^ We did not adjust for moderate-to-vigorous physical activity levels in the sedentary time analyses as suggested by Page *et al*,^59^ and given that in our study these measures were assessed simultaneously.

### Strengths and weaknesses of the study

This study was carried out using data from a large and contemporary UK cohort, using both cross-sectional and prospective study designs to explore concurrent and causal associations between PA/sedentary time and adiposity. Response weights and multiple imputation methods were also used to address attrition and missing data.

We used objective assessments of PA and sedentary time which, while overcoming the limitations of child or parental report, may underestimate activities not involving vertical movement of the trunk (such as cycling) and aquatic activities. We applied strict accelerometer data management criteria, including thresholds used to categorise intensity of activity and minimum required wear time, based on our previously published methodological studies.^36^ ^37^ ^39^ ^40^ As there was an interval between the interview at MCS4 and accelerometer assessments, the associations reported here are not truly cross-sectional. However in the longitudinal analyses the exposure (activity) and confounding factors were always measured before the outcome (adiposity), thus providing some support for a causal association.

We were able to take advantage of measures of fat mass obtained by trained interviewers using standardised protocols. The former are increasingly recognised as more appropriate measures of adiposity than BMI in young people,^18^ although many studies still only report BMI. The breadth of information recorded in MCS enabled consideration of a wide variety of potential confounding factors, including—importantly—an estimate of pubertal status. Unfortunately, information on dietary intake is limited in this cohort, reflecting in part the difficulties inherent in measuring energy intake and dietary quality reliably at this age. Ambrosini *et al*^60^ found longitudinal associations between dietary intakes characterised by energy density, % total energy from fat and fibre density and fat mass in children aged between 7 and 15 years, which they reported were independent of PA. However, this does not exclude an effect of moderate and/or vigorous physical activity since adjustment was for total PA only. We were unable to explore how this important factor may confound or mediate associations between PA and adiposity in our study. Further studies are needed that provide concurrent information on diet and objective assessment of PA.

Another strength of the MCS is its inclusion of children from ethnic minorities who—with the exception of the bi-ethnic Born in Bradford cohort^61^—are largely absent from other UK birth cohort studies. This enabled associations between activity and adiposity to be explored within different ethnic groups.

It is possible that children who are obese at age 7 years are less active as a consequence, thereby explaining the findings of the cross-sectional analyses.^62^ We adjusted for adiposity at age 7 years in the longitudinal analyses when these smaller but significant associations remained. A reduction in activity levels from 7 to 11 years, which we were unable to assess, may partially explain the reduction in effect sizes between the two study analyses. The lack of a second subsequent assessment of PA or sedentary time at or before age 11 years limits the extent to which PA trajectories could be taken into account in assessing the changes in adiposity. Objective assessments of activity are being repeated currently in cohort members at age 14 years and this will enable future analyses to examine changes in activity levels from childhood to adolescence and associations with subsequent adiposity.

### Implications for policy and practice

Our findings suggest that more active boys are at lower risk of subsequent adiposity. While our findings highlight the importance of promoting higher levels of PA, specifically of moderate-to-vigorous intensity level, in primary school-aged boys, as well as in girls who are known to be less active than boys. This is particularly important given evidence that transition to secondary school is associated with even lower activity levels.^63^ While increased PA is recognised to have a number of benefits, greater activity is generally promoted as part of a multifactorial approach to tackling childhood obesity, as evidenced by the recent WHO 2016 report which emphasises the global dimensions of childhood obesity.^4^ An increase in activity levels is likely to be particularly important for children from those ethnic groups at greater risk of obesity and its complications.^64^ However, efforts to increase activity levels in these groups need to reduce cultural and religious barriers, which have been shown to influence involvement.^65^

The case for policies regarding sedentary activities remain unclear: our findings suggest that this may make a less significant contribution to obesity risk however other evidence using proxy measures of sedentariness (eg, television viewing or screen time) provides some support for interventions aimed at re-allocating time from sedentary to active pursuits.
